# The Correlates of Government Expenditure on Mental Health Services: An Analysis of Data From 78 Countries and Regions

**DOI:** 10.7759/cureus.28284

**Published:** 2022-08-22

**Authors:** Ravi P Rajkumar

**Affiliations:** 1 Psychiatry, Jawaharlal Institute of Postgraduate Medical Education and Research, Pondicherry, IND

**Keywords:** mental health awareness, economic indicators, communicable diseases, culture, government funding, mental health

## Abstract

Background

Governmental investment in mental health is of vital importance for the implementation and maintenance of educational, preventive, and therapeutic services related to mental illness, particularly in low- and middle-income countries (LMICs). However, mental health expenditures represent only a small portion of total health spending in many countries. Little is known about the economic, social, or health-related factors that may influence variations in governmental spending in this sector.

Methods

Data on government expenditure on mental health as a percentage of total healthcare expenditure, collected by the WHO from 78 countries and regions in the period 2013-2014, was available for study. These data were analyzed in relation to key economic, social, and health-related indicators. The selection of these indicators was based on prior national and regional research and expert opinion as reported in the existing literature.

Results

Government spending on mental health was below 1% of health expenditure in 24.4% of the countries studied. A number of economic, social, and health-related indicators were significantly associated with variations in spending on mental health. Based on the partial correlation, sub-group, and multivariate linear regression analyses, the variables most significantly associated with low government spending on mental health were the burden of communicable diseases (β = −.47, *p *= .001) and cultural collectivism (β = −.37, *p *= .008).

Conclusions

These results suggest that low government investment in mental health may be associated not only with economic or political factors but also with variations in disease burden and in cultural attitudes across countries. Though no direct assumption regarding causation can be made, such findings may be of value when advocating for greater public investment in mental health, particularly in non-Western cultures with a high competing burden of infectious diseases.

## Introduction

Mental illnesses rank among the leading causes of global disease burden and disability. In the year 2019, mental illness was estimated to account for over 125 million disability-adjusted life years (DALYs) worldwide [1]. The findings of the Global Burden of Disease (GBD) studies suggest that the global and national disease burden caused by mental illness is increasing over time. For example, the proportion of global DALYs due to mental illness increased from 3.1% in 1990 to 4.9% in 2019, and the incidence of depression, which is the most commonly diagnosed mental illness, increased by almost 50% in the period 1990-2017 [1,2]. These figures represent a conservative estimate based on the available data and the use of certain statistical assumptions, whereas the true proportion of the global disease burden caused by mental illness may be significantly higher [3].

Despite the significant burden of disability and premature mortality associated with mental illnesses, there is often a discrepancy between the need for mental health services and their availability, particularly in low- and middle-income countries (LMICs). This phenomenon is sometimes referred to as the "treatment gap," referring to a deficit in the availability of medical treatments for mental illness, or the "care gap," a more inclusive term encompassing both medical and psychological treatments [4,5]. Though cost-effective medical and psychosocial interventions for mental illnesses exist and can bridge this "gap," their availability is often limited by a scarcity of human and material resources [6]. Moreover, even when services are available, affected individuals and their caregivers are often reluctant to access them due to the stigma surrounding mental illness in several cultures [7]. In order to successfully overcome the twin obstacles of stigma and scarcity of resources for mental health care, a combination of several factors is required: political will, planning, collaboration between different sectors of society, and adequate funding [6,8]. Funding is essential both for reducing stigma - through strategies such as community education and awareness campaigns - and for the provision of effective treatment in a sustainable manner [9]. In many cases, a large proportion of this funding is used for activities related to information, education, and communication in the sphere of mental health, with the aim of raising awareness and facilitating early diagnosis or even prevention [10]. Governmental investment is a key source of funding for mental health services in most countries, particularly when addressing the needs of under-served or underprivileged regions or sections of society [11,12]. However, there is often a substantial disparity between this investment and the actual burden of mental illness in many countries [13]. For example, a study comparing country-level mental health expenditures with estimated DALYs for mental, neurological, and substance-related disorders, covering 30 countries in the American region, found a median imbalance of 32:1 between disease burden and spending [14].

In 2001, the World Health Organization (WHO) collected nation-level information on the resources available for the treatment of mental illness, including details of budgetary allocation in 89 countries, as part of a wider initiative known as "Project Atlas." It was found that 36% of these countries allotted less than 1% of their health budget to mental health care, particularly in Africa and Southeast Asia [15]. Subsequent to the initial "Project Atlas," information on mental health spending in various countries was collected in the period 2013-2017, and this represents the most recently available data on this subject. These data were summarized in the WHO’s "Mental Health Atlas 2017," which provides region-wise information on mental health planning, resources, service availability, and mental health promotion or awareness activities [16]. As per this data, approximately two-thirds of countries have national programmes aimed at mental health promotion or preventive activities; the largest proportion of these were programmes aimed at improving mental health literacy. However, this report also noted that public spending on mental health in LMICs countries was low and was mostly used to fund in-patient psychiatric care (i.e., psychiatric hospitals or "asylums").

In the aforementioned study of 30 American countries, it was found that mental health spending was positively correlated with the national gross domestic product (GDP) [14]. However, there is evidence that other economic, social, and cultural variables may influence mental health spending. These include economic inequality; cultural attitudes that influence the stigma associated with mental illness; and variations between urban and rural regions within the same country [17-19]. Understanding these factors could aid the development of alternative strategies to address the gap between government spending and the actual burden of mental illness in a given population.

This study is an exploration of the associations between these data and certain key economic, cultural, and health-related variables, using the most recently available dataset (2013-2014) on government expenditure for mental health obtained from the World Health Organization’s database.

## Materials and methods

The current study is a cross-sectional, ecological analysis similar in nature to the earlier research of Vigo et al. on 30 countries on the American continent [14]. The aim of this study was to examine the strength, direction, and significance of the correlations between government spending on mental health and a wide range of socioeconomic and health-related variables. While the socioeconomic variables analyzed in this study were based on the work of Vigo et al. and extended to a larger number of countries [14], this research also aimed to build on these results by examining the impact of additional social and health-related variables. The study was primarily exploratory in intent; given its cross-sectional design, no direct inferences regarding causation or predictive power can be made.

Data sources

The World Health Organization’s Global Health Observatory (GHO) provides country-level data on several health-related indicators. For the purpose of this study, GHO data on government expenditure as a percentage of government expenditure on health - that is, the percentage of the health budget spent on mental health-related activities, whether educational, preventive, or therapeutic - was collected for a total of seventy-eight countries and territories. Information on this variable was collected during the period 2013-2014 based on responses provided to the WHO by member nations [20]. As this parameter represents the most recent estimate of government investment in mental health and was available for both high- and low-/middle-income countries, it was selected as the outcome or dependent variable for the purpose of this study.

Independent variables were selected based on two criteria: (a) direct or indirect evidence of an association between the given variable and government spending on mental health, based on the existing literature; and (b) availability of a reliable data source providing an estimate of the variable for the year 2014. Variables could be broadly classified into three categories: (a) economic indicators, (b) social and cultural indicators, and (c) health-related indicators.

A complete list of the variables selected for the analysis, the rationale for their inclusion, and the data source for each variable is provided in Table 1 [14,17,21-37].

**Table 1:** Variables studied in relation to government expenditure on mental health, with the rationale for their inclusion and data sources GDP: gross domestic product, EIU: Economist Intelligence Unit

Variable	Rationale for inclusion	Data source	Availability
Economic indicators			
GDP	GDP may be positively associated with mental health spending [14]	World Bank database [32] World Bank database [32]	76 countries
Healthcare spending, % of GDP	Some countries may allot a disproportionately low share of their healthcare budget to mental health care [21]		78 countries
Social spending, % of GDP	Higher social spending may be associated with better mental health outcomes and a lower need for mental health funding [22]		47 countries
Gini coefficient of economic inequality	Economic inequality may be associated with lower funding for mental health [17]	CIA World Factbook [33]	69 countries
Social indicators			
Democracy Index	Authoritarian governments may have a less positive attitude towards the funding of mental health services [23,24]	The EIU publication [34]	69 countries
Urbanization, % of population residing in urban areas	Spending on mental health may be higher in urbanized countries or regions [19]	World Bank database [32]	78 countries
Global Collectivism Index	Collectivist cultural values may be associated with greater mental health stigma and hence with lower funding [25,26]	Original research article [35]	72 countries
Infrastructure: number of hospital beds per 1,000 population	The capacity and sustainability of the healthcare system is may be associated with better funding for mental health [7]	World Health Organization Global Health Observatory database [36]	78 countries
Manpower: number of physicians per 1,000 population			78 countries
Illness-related indicators			
Burden of communicable diseases, estimated prevalence	In low- and middle-income countries, a high burden of communicable diseases may limit the availability of funds for mental health services [27]	Global Burden of Disease estimates, 2014 [37]	78 countries
Burden of non-communicable diseases (other than mental illness), estimated prevalence	Evidence for a strong association between mental illness and non-communicable diseases [28], but this is not reflected in planning or funding practices [29]		78 countries
Common mental disorders (anxiety and depression), estimated prevalence	Even in developed countries, funding for common mental disorders is often low when compared with the actual burden of these conditions [30]		78 countries
Severe mental disorders (bipolar disorder and schizophrenia), estimated prevalence	Mental health funding is often confined to the provision of in-patient care for severe mental disorders [16]		78 countries
National suicide rate, age-standardized per 100,000 population	Regional suicide rates may correlate with the allocation of funding for mental health services [31]		78 countries

Data analysis

All study variables were tested for normality prior to analysis. As none of the study variables conformed to a normal distribution (p < .01 for all variables, Shapiro-Wilk test), statistical analysis was carried out using the Mann-Whitney test for the comparison of medians between groups and Spearman’s rank correlation coefficient (ρ) for bivariate associations between variables.

For bivariate analyses, correlations between the percentage of healthcare spending allotted to mental health and each independent variable were examined. Due to the exploratory nature of this study, corrections for multiple comparisons were not attempted. All statistical tests were two-tailed, and the threshold for significance was set at p < .05. Assessments of the strengths of each bivariate correlation were made using the guideline values for biomedical research as follows: ρ ≥ 0.8, strong correlation; 0.6 ≤ ρ < 0.8, moderate correlation; 0.3 ≤ ρ < 0.6, fair correlation; ρ < 0.3, poor correlation [38]. If significant multicollinearity (ρ ≥ 0.8) was observed between any of the independent variables, partial correlation analyses were carried out to correct for any confounding effects caused by this phenomenon.

In an earlier study by Saxena et al., a value of 1% (i.e., 1% of government health spending allotted for mental health) was considered a minimum "acceptable" value for mental health funding by governments [15]. This cut-off was used in the current study for the purpose of group comparisons. While dividing countries into these two groups, differences between the medians of all the variables listed in Table 1 were examined. The purpose of this analysis was to identify the specific variables that differed between countries whose governments did or did not allot a minimum necessary portion of their healthcare budget for the specific purpose of mental health.

Finally, to identify those variables that were most strongly associated with mental health spending, a multivariate linear regression analysis was carried out. Owing to the small number of cases available for study and the degree of multicollinearity between certain variables, as described below, the following precautions were taken: (a) only variables showing at least a "moderate" correlation with mental health spending (ρ ≥ 0.6) were included in the analysis, (b) only variables with a plausible causal impact on mental health spending were selected, and (c) the stepwise method of regression was used.

## Results

Information on mental health expenditure as a percentage of total health expenditure (MH%) by governments was available for a total of 78 countries. These countries were distributed regionally as follows: African (n = 11), American (n = 19), Eastern Mediterranean (n = 6), European (n = 27), South-East Asian (n = 3) and Western Pacific (n = 12). When grouped according to the World Bank’s classification of countries based on income, the distribution of countries was: high income (n = 28), upper middle income (n = 24), lower middle income (n = 21), and low (n = 5). Overall, the median MH% was 2.79%, with an inter-quartile range of 3.87. Values ranged from a minimum of 0.1% in Zimbabwe to a maximum of 12.91% in France.

Comparisons of MH% based on region suggested that there was a significant variation overall between regions (Kruskal-Wallis H = 24.97, p < .001). However, on post-hoc comparisons, significant differences were observed only between the European and African regions (t = 4.00, p = .002) and between the European and American regions (t = 3.87, p = .003). Comparisons based on income group also found an overall difference (Kruskal-Wallis H = 32.51, p < .001). Post-hoc testing revealed that the high-income group differed significantly from all the other income groups: upper middle (t = 5.98, p < 0.001), lower middle (t = 5.78, p < .001), and lower (t = 4.39, p < .001). No significant differences in MH% were observed between the upper-middle, lower-middle, and low-income groups of countries.

When using the criterion suggested by Saxena et al. [15], it was observed that MH% was below 1% of the total health budget on mental health in 19 out of the 78 countries (24.36%). The 19 countries belonging to this category were Bangladesh, Burundi, Cameroon, the Dominican Republic, Gabon, Ghana, Haiti, Malawi, Malaysia, the Marshall Islands, Mexico, Mozambique, Nauru, Nepal, Palau, Papua New Guinea, Paraguay, Peru, and Zimbabwe. All these countries belonged to the low- and middle-income category; MH% below 1% was not observed in any high-income country.

A complete correlation matrix of the associations between MH% and the 14 independent variables examined in this study is presented in Table 2. MH% showed moderate positive correlations with the per capita GDP (ρ = .69, p < .001), Democracy Index (ρ = .61, p < .001), and the number of hospital beds and physicians per 100,000 population (ρ = .63 and .62, respectively, p < .001 in both cases). Fair positive correlations were observed for healthcare spending as a whole (ρ = .37, p < .001), urbanization (ρ = .44, p < .001), prevalence of non-communicable diseases (ρ = .33, p = .003) and prevalence of severe mental disorders (ρ = .52, p < .001). On the other hand, the prevalence of common mental disorders was only weakly correlated with MH% (ρ = .24, p = .032) and no correlation was observed between MH% and national suicide rates (ρ = .02, p = .883). MH% was moderately negatively correlated with the Gini coefficient (ρ = −.63, p < .001), Global Collectivism Index (ρ = −.77, p < .001) and prevalence of communicable diseases (ρ = −.78, p < .001).

**Table 2:** Correlation matrix of economic, social and illness-related variables associated with government funding for mental health CD: estimated prevalence of communicable diseases; CMD: estimated prevalence of common mental disorders; DI: Democracy Index; GCI: Global Collectivism Index; GDP: gross domestic product per capita; GINI: Gini coefficient of income inequality; HB: hospital beds per 100,000 population; HS: healthcare spending (% of GDP); MH%: mental health expenditure (% of total government health expenditure); NCD: estimated prevalence of non-communicable diseases; PHY: physicians per 100,000 population; SMD: estimated prevalence of severe mental disorders; SR: suicide rate per 100,000 population; SS: social spending (% of GDP), URB: percentage of population residing in urban areas. *Denotes a correlation that was significant at p < .05.

Variable	1 MH%	2 GDP	3 HS	4 SS	5 GINI	6 DI	7 URB	8 GCI	9 HB	10 PHY	11 CD	12 NCD	13 CMD	14 SMD	15 SR
1	-	.69*	.37*	.19	−.63*	.61*	.44*	−.77*	.63*	.62*	−.78*	.33*	.24*	.52*	.02
2		-	.37*	-.02	−.42*	.71*	.77*	−.86*	.56*	.76*	−.83*	.37*	.35*	.63*	.07
3			-	.29	−.23	.57*	.47*	−.53*	.34*	.42*	−.47*	.02	.31*	.37*	.18
4				-	−.23	.17	.09	−.11	.11	.17	−.21	.05	.03	.22	.24
5					-	−.37*	−.29*	.51*	−.65*	−.54*	.67*	−.45*	−.01	−.11	−.13
6						-	.60*	−.79*	.44*	.57*	−.65*	.22	.29*	.54*	.25*
7							-	-.69*	.43*	.57*	−.65*	.26*	.33*	.55*	−.04
8								-	−.69*	−.77*	.81*	−.44*	−.17	−.56*	−.28*
9									-	.75*	−.73*	.67*	−.16	.17	.11
10										-	−.85*	.48*	.15	.52*	.00
11											-	−.40*	−.30*	−.62*	.01
12												-	−.61*	−.14	.14
13													-	.59*	−.08
14														-	−.16

On examining the possibility of multicollinearity between independent variables, using a threshold of ρ ≥ .8, per capita GDP was negatively correlated with collectivism (ρ = −.86) and communicable disease prevalence (ρ = −.83); collectivism was positively correlated with communicable disease prevalence (ρ = .81); and communicable disease burden was negatively correlated with the number of physicians per 100,000 population (ρ = −.85). To correct for this, partial correlation analyses of these variables were carried out using Spearman’s partial correlations, as presented in Table 3. In these analyses, it was observed that the associations between MH% and per capita GDP and between MH% and the number of physicians per 100,000 population were no longer significant. The associations between MH% and the Global Collectivism Index and the estimated prevalence of communicable diseases remained statistically significant.

**Table 3:** Partial correlation analyses of variables associated with mental health expenditure by governments All values are given as Spearman’s partial ρ (significance level) *Denotes a partial correlation that was significant at p < .05

Variable	Uncorrected correlation with MH%	Corrected correlation with MH%	Variables corrected for
Gross domestic product per capita	.69 (.001)	−.01 (.993)	Global Collectivism Index, estimated prevalence of communicable diseases
Global Collectivism Index	−.77 (.001)	−.39 (< .001)*	GDP per capita
Number of physicians per 100,000 population	.62 (.001)	−.14 (.230)	Estimated prevalence of communicable diseases
Estimated prevalence of communicable diseases	−.78 (.001)	−.45 (< .001)*	GDP per capita, Global Collectivism Index, number of physicians per 100,000 population

Comparisons between countries with MH% < 1 and MH% ≥ 1 are presented, along with their significance and effect size, in Table 4. In this analysis, the per capita GDP, Democracy Index, number of hospital beds and physicians per 100,000 population, and the prevalence of non-communicable diseases and severe mental disorders were all significantly lower in countries where MH% was below 1. On the other hand, these countries had a significantly higher Gini coefficient, Global Collectivism Index, and prevalence of communicable diseases. The effect size for all of these differences fell between 0.5 and 0.8, indicating a medium effect size.

**Table 4:** Comparisons of economic, social and illness-related indicators between countries where government expenditure on mental health is <1% of health spending and those where expenditure is ≥ 1% GDP, gross domestic product; IQR, inter-quartile range

Variable	Median (IQR) (MH% <1)	Median (IQR) (MH ≥ 1)	Mann-Whitney test statistic	Significance level	Effect size
Gross domestic product	3653.00 (8850.50)	18661.00 (27180.00)	207.0	.001	−0.62
% of GDP spent on health	5.43 (4.05)	6.96 (3.47)	416.0	.094	−0.26
Social spending, % of GDP	1.16 (1.06)	1.37 (1.51)	172.5	.126	−0.28
Gini coefficient of income inequality	43.15 (6.63)	34.4 (9.10)	637.5	.002	0.50
Democracy Index	5.69 (2.68)	6.63 (2.73)	265.0	.024	−0.38
Urbanization, % of population living in urban areas	52.16 (43.39)	67.96 (29.86)	426.5	.120	−0.24
Global Collectivism Index	0.63 (0.57)	-0.21 (1.18)	732.5	.001	0.64
Hospital beds per 100,000 population	9.80 (6.36)	28.62 (27.27)	103.5	.001	−0.75
Physicians per 100,000 population	3.84 (11.93)	23.72 (20.49)	171.0	.001	−0.70
Communicable diseases, estimated prevalence	68.69 (11.49)	45.77 (26.53)	997.0	.001	0.78
Non-communicable diseases, estimated prevalence	81.71 (2.24)	82.30 (3.32)	348.5	.014	−0.38
Common mental disorders, estimated prevalence	7.97 (1.06)	8.50 (3.00)	489.0	.409	−0.13
Severe mental disorders, estimated prevalence	0.77 (0.28)	1.11 (0.41)	246.5	.001	−0.56
Suicide rate per 100,000 population	9.00 (4.13)	7.90 (7.83)	517.5	.350	0.16

The results of the multivariate regression analysis, taking MH% as the dependent variable, are presented in Table 5. Of the five variables selected for inclusion in the analysis - GDP per capita, Gini coefficient, Democracy Index, Global Collectivism Index, and prevalence of communicable diseases - only two were retained as significant in the final model: prevalence of communicable diseases (β = −.47, p = .001) and the Global Collectivism Index (β = −.37, p = .008). Variance inflation factors were below 4 for both variables, indicating that a significant degree of multicollinearity was unlikely to have affected the results.

**Table 5:** Stepwise multivariate linear regression analysis of the variables associated with government expenditure on mental health

Variable	Regression coefficient (β)	Significance level	Variance inflation factor
Estimated prevalence of communicable diseases	−.47	.001	3.24
Global Collectivism Index	−.37	.008	3.24

## Discussion

Mental disorders constitute a significant proportion of the global disease burden; however, the coverage and accessibility of mental health care continue to lag behind the prevalence of these disorders. Mental health care should not be seen as confined to the provision of in-patient beds and medications for severe mental illness; it encompasses a wide range of activities related to mental health promotion, education, reduction of stigma, and the provision of treatment both in hospitals and in community settings [5-9]. In many low- and middle-income countries, patients with mental disorders have to pay for their own care in private settings, and the quality of public mental health care remains poor due to the low priority accorded to funding for mental health [39]. While it seems a straightforward proposition to advocate for increased public funding for mental health, such advocacy requires an understanding of the factors that influence the extent of such funding at a regional or national level. This study aims to contribute to such advocacy through a provisional identification of factors that are associated with cross-national variations in mental health funding.

In the current analysis, several variables were associated with the percentage of government health spending allotted to mental health. These findings are consistent with the results of prior research and expert opinion, suggesting that mental health spending was strongly associated with measures of economic prosperity and inequality [14,17], with the general level of health infrastructure in a given country [7], with the type of government [24], with cultural values that could influence public perception of mental disorders [25,26,40], and with the pattern of disease burden in a given country.

Given the substantial correlations between some of these variables themselves, no clear assumptions can be made regarding causality. For example, bivariate analyses showed a strong correlation between GDP and mental health spending, but this was not significant after correcting for potential confounders (Table 3). While it is true that all the countries with a "low" MH% value belonged to the low- and middle-income category, there was substantial variation within this group, and some countries classified as low- or middle-income, such as Algeria (7.5%) and Tunisia (5.0%), allotted a substantial proportion of their health expenditure to mental health. Likewise, in some countries classified as upper-middle-income, such as Malaysia (0.4%) and Palau (0.8%), government expenditure on mental health was lower than in low-income countries such as Eritrea (1.9%) and Malawi (1%). This was corroborated by the analysis of MH% by income group, which found a significant difference only between high-income countries and the remainder. Economic inequality, as measured by the Gini coefficient, was also associated with lower levels of government investment in mental health, but this association was not significant in the multivariate analysis, suggesting that it may reflect the effects of cofounding factors.

Countries with a more authoritarian form of government, as indicated by a lower Democracy Index, had lower levels of government funding for mental health. This phenomenon has been demonstrated historically in countries such as Argentina, where a shift from a more democratic government to a military dictatorship was associated with an abrupt cessation of collaboration between the government and mental health professionals [24]. Change in the opposite direction has also been documented, as in the case of the Netherlands [41]. The formulation of a coherent and far-reaching mental health policy, involving the perspectives of multiple stakeholders, is essentially a democratic process, and such a process may be hindered by authoritarianism or factionalism [42]. Authoritarianism may also be associated with increased criminalization of mental illness and related conditions, such as substance use, leading to an emphasis on punitive rather than therapeutic measures [43]. However, caution is required in interpreting this result as the association with the Democracy Index was not significant in the multivariate regression analysis.

When considering all the variables examined in this study, two factors remained significantly associated with mental health spending: the prevalence of communicable diseases and the Global Collectivism Index. The strong inverse correlation between MH% and the burden of communicable diseases is consistent with the observations of McDaid et al., who noted that in lower-income countries with a high burden of disease morbidity and mortality caused by infectious diseases, these conditions may be given a higher priority than mental disorders [27]. This finding is significant, as it suggests that reduced investment in mental health may not be solely the result of economic or political factors, but of public health problems that are perceived, both by the public and by governments or other funding agencies, as more serious and requiring more urgent intervention [44,45]. Furthermore, interventions for the prevention and treatment of infectious diseases are more easily understood by non-specialists and are seen as cost-effective and time-limited, in contrast with interventions related to mental disorders, which are poorly understood by many and seen as time-consuming and ineffective [46]. This is compounded by the difference between the immediate benefits derived from communicable disease control, which are easily understood, and the delayed and often "hidden" benefits of mental health care [27,39]. There are also practical difficulties in communicating contemporary models of mental illness and their treatment, particularly in Eastern cultures, whereas concepts such as contagion and vector control are relatively easy to understand across cultures [27,47]. Correcting this inequality would require the development of awareness about the economic benefits of treating mental disorders [27], a deeper understanding of the link between mental disorders and infectious diseases [47], and the promotion of effective mental health awareness programmes and interventions at the community level, to dispel misconceptions related to the efficacy or cost of such interventions [48]. It should also be emphasized to both the public and policymakers that appropriate investment in mental health can improve outcomes for both patients and their caregivers [49], while a lack of such investment can lead to worse outcomes from both health-related and economic perspectives [50].

The association between cultural collectivism and lower government spending, identified in the multivariate analysis, can be understood at several levels. Collectivist cultures are associated with an expectation of conformity to social norms, which often cannot be met by patients with mental disorders. This perception of a violation of social norms leads to higher levels of shame and stigma attached to these disorders, which would be reflected in the attitude of local and national governments [51]. There was a strong negative correlation between cultural collectivism and the Democracy Index in the current dataset (ρ = −.79, p < .001). This link between cultural values and an authoritarian form of government may also partly explain the link between this variable and lower government investment in mental health [43]. There is a relative paucity of empirical research on the links between individualism and collectivism, or other cultural value dimensions, and public or governmental attitudes towards mental illness, suggesting that this relationship requires further exploration [51].

The current study has certain significant limitations. First, it is based on data collected during the period 2013-14, and it is likely that at least some governments may have subsequently increased their level of expenditure on mental health. Second, it is largely based on data from high- and middle-income countries, implying that these results may vary if data on a larger number of low-income countries was available. Third, due to its cross-sectional nature, no inference regarding causative mechanisms can be drawn from this study; answering such questions (for example, "does cultural collectivism cause lower government investment in mental health through a link with political authoritarianism?") would require a larger data set, longitudinal data collection, and more sophisticated methods of statistical analysis. Fourth, owing to a relative paucity of research in this area, the selection of variables for analysis was based on a small number of studies; it is possible that other variables of interest may have been inadvertently omitted. Fifth, as the analysis in this study is at the national level, it cannot provide insights into the factors associated with variations in mental health spending within a country, such as urban-rural inequalities. Finally, it should be clearly stated that government investment in mental health is only a first step - albeit a crucial one - in developing effective and sustainable services for the promotion of mental health awareness and the treatment of mental disorders. Such programmes require planning, political will, cooperation between different sections of society, transparency, and efficient systems for the implementation of recommendations at the community level [52].

## Conclusions

Despite evidence of a modest increase in government expenditure on mental health in the period 2003-2013, the current study’s findings suggest that the level of government investment in this domain of health remains sub-optimal in a significant minority of countries. Reduced government investment in mental health may not be the result of economic or political factors alone but may reflect the influence of cultural or disease-related factors which should be addressed in their own right. It is hoped that these findings will be of use in analyzing and interpreting more recent data on government expenditure in this field and in the development of models that could identify the factors facilitating or hindering such spending in a more robust manner. These findings require replication across a wider range of countries covering different geographical locations and cultures, which would be facilitated by the inclusion of a larger number of countries in subsequent editions of the WHO Mental Health Atlas and related projects. The wider dissemination of such data and research findings would help both health professionals and policymakers in planning, funding, and providing mental health services that are culturally appropriate, cost-effective, and acceptable to the general public.
